# Optimization and Experimental Analysis of Electroless Nickel Plating on the Diamond Surface

**DOI:** 10.3390/mi16060709

**Published:** 2025-06-13

**Authors:** Qingming Fan, Guokang Su, Congmin Zhu, Hui Qi, Pengfan Li, Xiumei Shen, Chuanyun Zhang, Kai Cheng

**Affiliations:** 1 School of Mechatronic Engineering, Xi’an Technological University, Xi’an 710021, China; fanqingming@xatu.edu.cn (Q.F.); suguokang@xatu.edu.cn (G.S.); zhucongmin7@gmail.com (C.Z.); lpf9697@163.com (P.L.); shenxiumei@xatu.edu.cn (X.S.); 2 Shaanxi Engineering Research Center of Digital Precision Electrochemical Machining, Xi’an 710021, China; 3 College of Chemistry and Materials, Taiyuan Normal University, Jinzhong 030619, China; qihui@xatu.edu.cn; 4 College of Engineering, Design and Physical Sciences, Brunel University London, London UB8 3PH, UK; kai.cheng@brunel.ac.uk

**Keywords:** diamond surface, electroless plating nickel, AgNO_3_, activator

## Abstract

Coating diamond particle surfaces with a layer of high-temperature resistant nickel, which possesses weldability, effectively enhances the bonding strength between diamond particles and substrates in pre-grinding tools. This improves their stability and strength at high temperatures, thereby enhancing the performance, lifespan, and efficiency of grinding tools. This paper explores the electroless nickel plating process on diamond surfaces, analyzes the working principle of electroless nickel plating on diamond surfaces, and proposes the use of 2 g/L AgNO_3_ solution and 2 g/L AgNO_3_ + 10 mL/L NH_3_·H_2_O solution as Pd-free activating solutions. Experimental studies have demonstrated the feasibility of using silver nitrate as an activator, and it has been found that the 2 g/L AgNO_3_ + 10 mL/L NH_3_·H_2_O solution achieves a higher surface plating ratio when used as an activator for electroless nickel plating on diamond surfaces. Based on this, through orthogonal and single-factor experimental methods, the effects of ammonia solution concentration, sodium hypophosphite concentration, plating temperature, and diamond particle size on electroless nickel plating on diamond surfaces were investigated. The optimal process for electroless nickel plating on diamond surfaces was obtained: ammonia solution concentration of 17.5 mL/L, sodium hypophosphite concentration of 33 g/L, and plating temperature of 80 °C. Under this process, using diamond particles with a size of 120/140 for electroless nickel plating, a surface plating ratio of 10.75% electroless nickel-plated diamond can be achieved.

## 1. Introduction

Diamonds are known as the hardest material in nature [1] and possess exceptional physical, chemical, and mechanical properties, such as superior thermal conductivity [2], good chemical stability [3], and an extremely high melting point [4]. These characteristics endow diamonds with significant application value in the industrial sector. Generally, diamond particles in diamond tools are integrated with the matrix through binder or mechanical embedding methods. However, under conditions of high-speed and high-pressure grinding forces, these integration methods may not effectively ensure a robust bond between the diamond particles and the matrix. At high temperatures, diamonds are prone to oxidation and weight loss, and may even undergo graphitization, leading to a decrease in their compressive strength and subsequently affecting the efficiency of diamond tools. Therefore, enhancing the bonding strength between diamond particles and the matrix, while simultaneously improving the stability of diamond particles under high-temperature environments, has become one of the research focuses.

Research has found that by coating diamond particles with a layer of metal, the bonding strength between the diamond particles and the matrix in diamond tools can be effectively enhanced. This, in turn, improves their stability and strength at high temperatures, thereby extending the service life, performance, and efficiency of the diamond grinding tools [5,6,7,8]. The most commonly used method involves coating the surface of diamond particles with a layer of metallic material through physical or chemical means, encapsulating the diamond particles and thus enhancing their bonding strength with the matrix, as well as improving their stability at high temperatures. Currently, commonly used diamond surface coating methods include electroless plating [9], physical vapor deposition (PVD) [10], chemical vapor deposition (CVD) [11], electroplating [12], vacuum micro-evaporation coating, and salt bath plating [13]. Compared to other methods, the electroless plating process does not require an external electrical current and can efficiently produce uniform coatings that cover even complex surfaces.

In electroless plating, the use of palladium as an activator is not only costly but also leads to certain precious metal pollution. Therefore, there is a need to explore new palladium-free activation processes for diamond electroless plating. Tian et al. [14] developed a palladium-free activation method for electroless nickel plating on copper surfaces through immersion nickel technology. It demonstrated that the catalytic activity of this Ni-activation method was slightly lower as compared to the conventional Pd-activation, but both obtained electroless Ni–P layers exhibited similar morphology, chemical composition, corrosion resistance, and adhesion strength. Luo et al. [15] proposed a new available palladium-free surface activation process on polycarbonate (PC) engineering plastic before electroless plating. The growth mechanisms were analyzed as the reactant (Cu^2+^) in the electroless plating solution dispersed and were adsorbed by the plastic surface in the process of Cu plating. Shu et al. [16] investigated an environment-friendly surface etching and activation techniques for acrylonitrile–butadiene–styrene (ABS) surface metallization as a replacement for conventional chromic acid etching bath and palladium catalyst. After etching by H_2_SO_4_-MnO_2_ colloid, the ABS surfaces became rough; meanwhile the carboxyl and hydroxyl groups were formed on the surface. Currently, there is still relatively little research on the palladium-free activation chemical plating process for diamonds.

Metal nickel possesses characteristics such as high temperature resistance, resistance to oxidation, excellent mechanical strength, ductility, and corrosion resistance, making it commonly used as a material for diamond surface coating.

Currently, the most commonly used method for nickel plating on diamond surfaces is chemical nickel plating [17,18,19]. Typically, chemical nickel plating on diamond surfaces is subdivided into two steps: diamond surface pretreatment and chemical nickel plating. And the key to the pretreatment lies in the activation process. This article employs silver nitrate as an activator for chemical nickel plating on diamond surfaces, replacing palladium chloride, which is expensive and environmentally harmful, and verifies its effectiveness.

## 2. Methods and Principles

**Figure 1:**
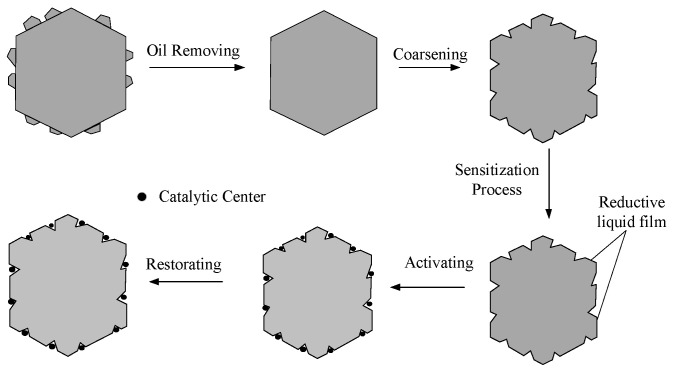
Process flow diagram for diamond surface pretreatment.

Due to the absence of catalytic centers on the diamond surface, electroless nickel plating cannot proceed directly. Therefore, prior to electroless nickel plating, it is necessary to pretreat the diamond surface to deposit some metal particles as catalytic centers on its surface. Subsequently, electroless nickel plating can be conducted. As illustrated in Figure 1, it describes the process flow of diamond surface pretreatment, which includes the following steps in sequence: surface degreasing, roughening, sensitization, activation, and reduction. The diamond surface is pretreated to obtain a pitted surface. Within these pits, metal particles are deposited, serving as catalytic centers on the diamond surface.

**Figure 2:**
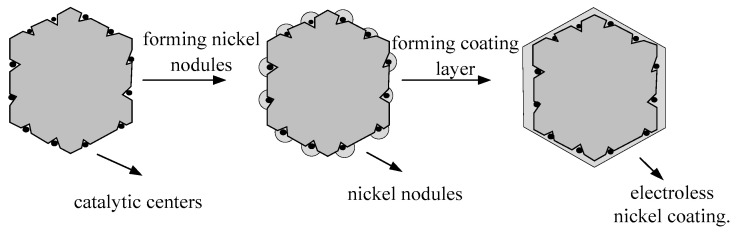
Schematic diagram of the growth model of electroless nickel plating.

Electroless nickel plating on the surface of the diamond involves dehydrogenation at the catalytic centers on the diamond surface through hypophosphite ions in the solution. Nickel ions are reduced to nickel monoxide under the influence of hydrogen atoms, subsequently depositing on the surface of the catalytic centers and gradually forming nickel nodules. These nickel nodules on the diamond surface act as catalytic centers, enabling the deposition of newly reduced nickel monoxide around them. As the electroless nickel plating reaction continues, a continuous and dense coating layer is gradually formed on the diamond surface. Figure 2 illustrates the growth model of the electroless nickel coating.

The electroless nickel plating on the surface of the diamond primarily involves the following processes:

The hypophosphite ion undergoes dehydrogenation under the catalysis of the catalytic center on the diamond surface, resulting in the formation of phosphite ions and hydrogen atoms. The generated hydrogen atoms adsorb onto the surface of the catalytic center. The chemical reaction is depicted in Equation (1).

(1) $$H_{2}{\mathrm{PO}}_{2}^{-}{\;+\;H}_{2}O\to H^{+}{\;+\;\mathrm{HPO}}_{3}^{2-}\;+\;2H$$

Hydrogen atoms adsorbed on the surface of the catalytic center reduce nickel ions in the electroless plating solution to metallic nickel, which then deposits on the surface of diamond particles. The chemical reaction is depicted in Equation (2).

(2) $${\mathrm{Ni}}^{2+}\;+\;2H\to {\mathrm{Ni}\;+\;2H}^{+}$$

Certain hypophosphite ions are also adsorbed onto the surface of the catalytic center and reduced to elemental phosphorus by the active hydrogen atoms, as depicted in Equation (3).

(3) $$H_{2}{\mathrm{PO}}_{2}^{-}\;+\;H\to {P\;+\;H}_{2}{O\;+\;\mathrm{OH}}^{-}$$

In the process of electroless nickel plating, besides the reduction reaction of nickel ions, a hydrogen evolution reaction also occurs, as shown in Equation (4).

(4) $$H_{2}{\mathrm{PO}}_{2}^{-}\;+\;H_{2}O\to H^{+}{\;+\;\mathrm{HPO}}_{3}^{2-}{\;+\;H}_{2}↑$$

## 3. Selection of Activating Agents

Activation is a crucial step in the pretreatment of diamond surfaces. Typically, palladium chloride solution is employed as the activator; however, it is expensive and environmentally harmful. Silver, a transition metal element, possesses atomic structural characteristics akin to palladium, both containing unfilled d-orbitals. These unfilled d-orbitals provide active sites conducive to interacting with reactant molecules. This interaction can lower the activation energy of the reaction, thereby enhancing catalytic activity. Consequently, silver can serve as the catalytic center on the diamond surface. In this paper, a silver nitrate solution with a concentration of 2 g/L is selected as the activator in the pretreatment of diamond surfaces, replacing palladium chloride.

The activating solution composed of AgNO_3_ and deionized water exhibits poor stability in the atmosphere. When this activating solution is utilized for the activation of diamond particles, a portion of it decomposes during the activation process, thereby diminishing the effectiveness of the activation. The decomposition reaction is represented by Equation (5).

(5) $${2\mathrm{AgNO}}_{3}\to {2\mathrm{Ag}\;+\;2\mathrm{NO}}_{2}↑\;+\;O_{2}↑$$

Therefore, the activation of the diamond surface using an activating solution composed of AgNO_3_ and deionized water results in fewer active centers on the activated diamond surface, which in turn leads to a lower surface plating ratio of electroless nickel on diamond. The activation reaction mechanism is illustrated in Equation (6).

(6) $${2\mathrm{Ag}}^{+}{\;+\;\mathrm{Sn}}^{2+}\to {\mathrm{Sn}}^{4+}\;+\;2\mathrm{Ag}↓$$

To effectively enhance the stability of the activating solution, a concentration of 10 mL/L NH_3_·H_2_O was added to a 2 g/L silver nitrate activating solution. The NH_4_^+^ provided by ammonia solution serves as additional coordination bonds for AgNO_3_, inhibiting the reduction of silver ions, thereby enhancing the stability of the solution, preventing self-decomposition of the activating solution, and improving the activating effect. The reaction equation for the silver–ammonia complexation reaction is as follows, shown in Equation (7):

(7) $${\mathrm{AgNO}}_{3}\;+\;3{\mathrm{NH}}_{3}\cdot H_{2}O\to \;\mathrm{Ag}\;{\left({\mathrm{NH}}_{3}\right)}_{2}\mathrm{OH}\;+\;2H_{2}O\;+\;{\mathrm{NH}}_{4}{\mathrm{NO}}_{3}$$

The mechanism of the activation reaction is illustrated in Equation (8):

(8) $$2{\left[\mathrm{Ag}{\left({\mathrm{NH}}_{3}\right)}_{2}\right]}^{\;+\;}{+\mathrm{Sn}}^{2+}\to {\mathrm{Sn}}^{4+}\;+\;2\mathrm{Ag}↓\;+\;{4\mathrm{NH}}_{3}$$

Regarding the inclusion of ammonia solution in silver nitrate, two activation solutions were proposed, with their chemical compositions shown in Table 1. The study investigated the effects of these two activation solutions during the electroless nickel plating process on diamonds, selecting the activation solution with superior activation performance.

**Figure 3:**
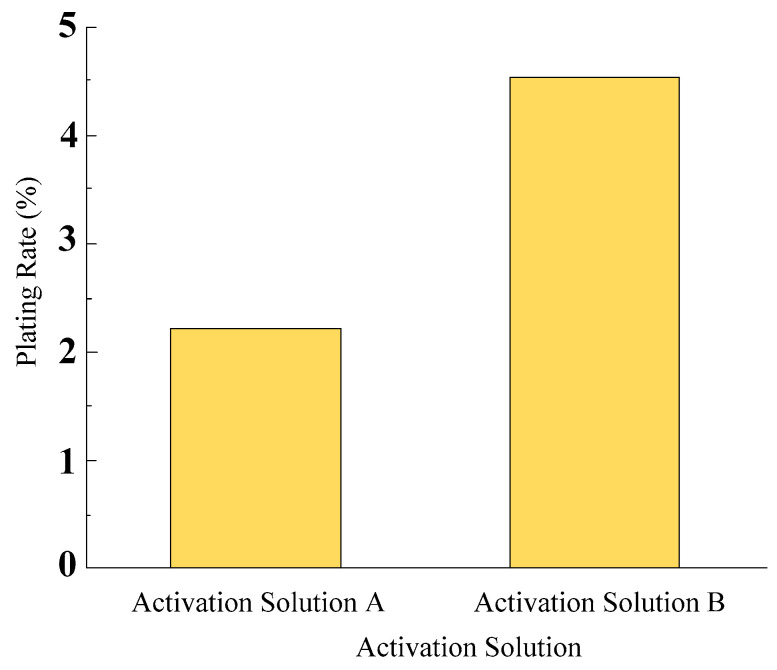
Surface plating ratio of electroless nickel plating on diamonds under different activation solutions.

Diamonds with a particle size of 45/50 were activated using Activation Solution A and Activation Solution B. Under the conditions of a plating temperature of 80 °C and a rotational speed of 100 r/min with magnetic stirring, the pretreated diamond particles underwent electroless nickel plating for 30 min. Following the completion of electroless nickel plating, the nickel-plated diamonds were cleaned, dried, weighed, and subjected to scanning electron microscopy analysis. As shown in Figure 3, which illustrates the surface plating ratios of electroless nickel-plated diamonds under different activation solutions, it can be observed that the use of Activation Solution B for diamond activation results in a higher surface plating ratio of electroless nickel-plated diamonds.

**Figure 4:**
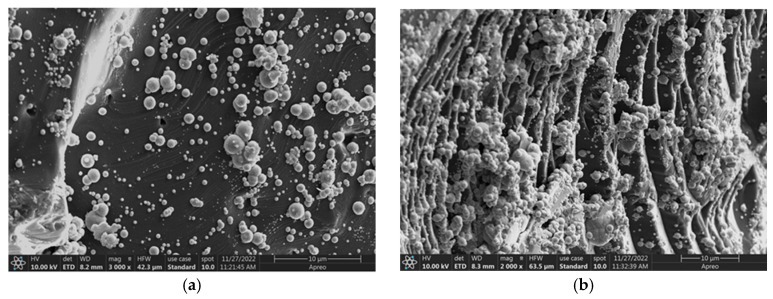
Scanning electron microscopy images (×3000) of electroless nickel plating on diamond coatings under different activating solutions. (**a**) Activation Solution A, (**b**) Activation Solution B.

Figure 4 illustrates scanning electron microscopy images of electroless nickel-plated diamond coatings under various activating solutions. As evident from panels (a) and (b), nickel nodules have formed on the diamond surface, with dimensions that are essentially uniform. However, a comparative analysis of the number of nickel nodules generated on the diamond surface reveals that the use of Activation Solution B as the activator results in a higher number of nickel nodules on the surface of the electroless nickel-plated diamond, indicating that Activation Solution B exhibits superior activating effects compared to Activation Solution A.

## 4. Experimental Design

### 4.1. Composition of Electroless Nickel Plating Solution

The electroless nickel plating solution utilized comprises primarily the following components: metal salt, reducing agent, complexing agent, stabilizer, and dispersant. The composition and function of each component in the plating solution are outlined in Table 2 [20,21].

(1) Weigh an appropriate amount of citric acid and dissolve it in 50 mL of ultrapure water. Continue heating and stirring until the solution becomes transparent. Subsequently, add an appropriate amount of sodium citrate, heat and stir until dissolved to obtain a transparent solution, designated as solution a.
(2) Weigh an appropriate amount of NiSO_4_·6H_2_O and dissolve it in 50 mL of ultrapure water to obtain solution b. Subsequently, gradually add solution a to solution b, resulting in solution c.
(3) Weigh an appropriate amount of sodium hypophosphite and dissolve it in 50 mL of ultrapure water, stirring continuously to ensure complete dissolution, thereby obtaining solution d. Subsequently, add solution d to solution c and heat while stirring to dissolve, resulting in solution e.
(4) Add an appropriate amount of ammonia solution to solution d, continue heating and stirring until complete dissolution is achieved. Subsequently, introduce an adequate quantity of Sodium Dodecyl Benzene Sulfate (SDBS) and thiourea, and stir for 5 min to obtain the electroless plating solution f.

### 4.2. Design of the Experimental Scheme

From the chemical reaction Equations (1) and (2), the following can be observed: Firstly, during the electroless nickel plating process, H^+^ is continuously generated, leading to a continuous decrease in the pH value of the solution. However, upon the addition of ammonia solution, the H^+^ in the solution is neutralized, and the chemical reaction proceeds in the positive direction. Therefore, the concentration of ammonia solution in the plating solution is crucial for the electroless nickel plating reaction on the diamond surface. Secondly, during the electroless nickel plating process, nickel ions are reduced to nickel metal by the action of active hydrogen, depositing on the diamond surface to form a plating layer.

The role of sodium hypophosphite in the electroless nickel plating process is to provide the active hydrogen required for the reduction of nickel ions. Its concentration determines the content of active hydrogen in the solution, which in turn affects the reduction rate and plating efficiency of nickel ions. Sodium hypophosphite decomposes to release active hydrogen at a certain temperature. A higher plating temperature aids in accelerating the decomposition rate of sodium hypophosphite, increasing the content of active hydrogen in the solution, and promoting the reduction reaction of nickel ions, thereby facilitating the electroless nickel plating process. Conversely, if the plating temperature is too low, it may lead to a slower decomposition of sodium hypophosphite and insufficient active hydrogen, affecting the nickel reduction process, resulting in a decrease in plating quality or abnormal plating solution reactions. Therefore, during the electroless nickel plating process, it is necessary to control the plating temperature to ensure that sodium hypophosphite can decompose sufficiently, releasing enough active hydrogen to ensure normal plating solution reactions.

Based on the comprehensive analysis, it can be concluded that the concentration of ammonia solution and sodium hypophosphite in the plating solution, as well as the plating temperature during the electroless nickel plating process, exert significant impacts on the electroless nickel plating on the diamond surface. A three-factor, three-level orthogonal experiment was designed, as presented in Table 3, to investigate the effects of ammonia solution concentration, sodium hypophosphite concentration, and plating temperature during the electroless nickel plating process on the electroless nickel plating solution.

### 4.3. Analysis of Preliminary Experimental Results

Based on the orthogonal experiment designed in Section 4.2, various experimental schemes were tested and calculated; the results are shown in Table 4.

Table 5 was obtained through the application of range analysis. Based on the experimental results, the primary and secondary relationships of the impact of various influencing factors on the deposition surface plating ratio of electroless nickel plating on diamonds can be determined as follows: plating temperature > ammonia solution concentration > sodium hypophosphite concentration. The optimal parameter combination for deposition rate is as follows: ammonia solution concentration of 17.5 mL/L, sodium hypophosphite concentration of 33 g/L, and deposition temperature of 80 °C. Under the condition that other factors remain unchanged, using this set of parameters, the deposition rate of electroless nickel plating on diamond can reach 8.18%. In Table 5, R1, R2, and R3 represent the average values of the sum of experimental results corresponding to factors 1, 2, and 3, respectively.

## 5. Results and Discussion

### 5.1. The Influence of Ammonia Concentration on Electroless Nickel Plating

A single-factor experiment was designed to investigate the influence of ammonia concentration in the electroless nickel plating solution on the deposition rate and microstructure of the electroless nickel-coated diamond. The plating temperature was set at 80 °C, the diamond particle size was 45/50, the diamond loading was 1 g, the magnetic stirring speed was 100 r/min, the electroless nickel plating duration was 60 min, and the ammonia concentration ranged from 12.5 to 20 mL/L. The plating solution parameters with different ammonia concentration are presented in Table 6.

#### 5.1.1. The Influence of Ammonia Concentration on the Surface Plating Ratio

**Figure 5:**
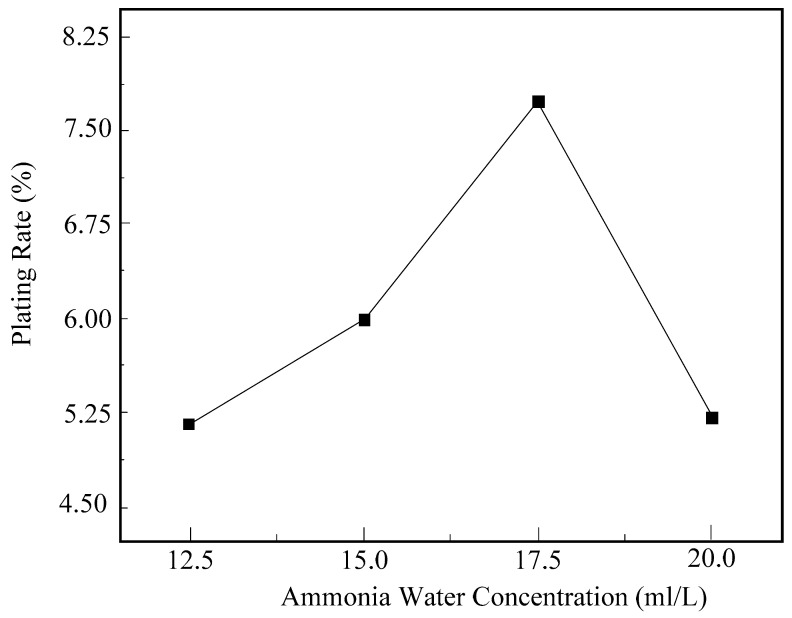
The influence of ammonia solution concentration on the surface plating ratio.

Figure 5 illustrates the impact of ammonia solution concentration on the surface plating ratio. The graph reveals that when the ammonia solution concentration in the solution is 12.5 mL/L, the surface plating ratio is comparatively low. The primary reason for this is that at lower ammonia solution concentrations, the pH value of the electroless plating solution is lower, and the reducing capability of the solution is weaker. Consequently, less Ni^2+^ is reduced, leading to a lower surface plating ratio. As the ammonia solution concentration increases, the pH value of the electroless plating solution also rises, rapidly reducing the H^+^ concentration in the solution. This shift in pH favors the forward direction of the reaction, resulting in a gradual increase in the surface plating ratio.

When the concentration of ammonia solution increased to 17.5 mL/L, the surface plating ratio reached its maximum. However, as the concentration of ammonia solution continued to rise to 20 mL/L, the surface plating ratio began to decrease. The primary reason for this is that, with the increasing concentration of ammonia solution, the reaction rate of electroless nickel plating becomes excessively rapid. Consequently, the reduced nickel element fails to deposit on the diamond surface and instead forms nickel slag, precipitating at the bottom of the beaker, thereby leading to a reduction in the surface plating ratio.

#### 5.1.2. The Influence of Ammonia Concentration on the Microscopic Morphology of the Coating

**Figure 6:**
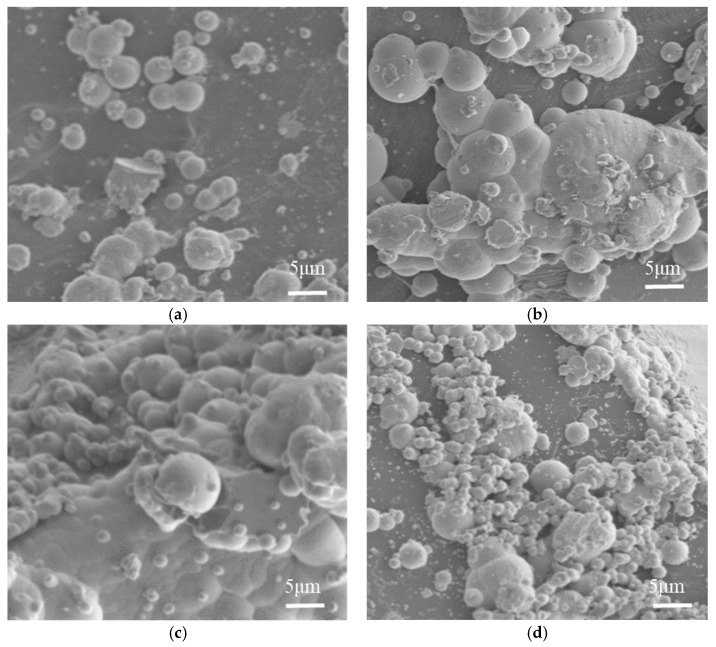
Scanning electron microscopy images (×1000) of the microscopic morphology of the coating at different concentrations of ammonia solution. (**a**) Ammonia solution concentration of 12.5 mL/L, (**b**) ammonia solution concentration of 15.0 mL/L, (**c**) ammonia solution concentration of 17.5 mL/L, (**d**) ammonia solution concentration of 20.0 mL/L.

Scanning electron microscopy was employed to observe the microtopography of nickel-plated diamond coatings under varying ammonia concentrations, with the results presented in Figure 6. As found in Figure 6a, when the ammonia concentration is low, the diamond surface exhibits only a small number of nickel nodules with relatively small sizes, indicating no significant plating formation. With increasing ammonia concentration, as shown in Figure 6b,c, the diamond surface generates an increasing number of nickel nodules with progressively larger sizes. When the ammonia concentration reaches 17.5 mL/L, a distinct plating layer is formed on the diamond surface. Furthermore, as depicted in Figure 6d, as the ammonia concentration continues to rise to 20 mL/L, the plating becomes uneven, accompanied by significant underplating, and the size of the nickel nodules begins to decrease.

At lower concentrations of ammonia, the reaction rate is slow, preventing the formation of a coating. As the ammonia concentration gradually increases, the reaction rate accelerates, leading to an increasing number and size of nickel nodules on the diamond surface, and the formation of a distinct coating. However, when the ammonia concentration is excessively high, the reaction rate becomes too rapid, resulting in the partial reduction of nickel ions in the solution. This leads to the precipitation of nickel slag at the bottom of the beaker and the deposition of some on the diamond surface, causing missed plating.

### 5.2. The Influence of Sodium Hypophosphite Concentration on Electroless Nickel Plating

A single-factor experiment was designed to investigate the influence of sodium hypophosphite concentration in the electroless nickel plating solution on the deposition rate and microstructure of the electroless nickel-coated diamond. The plating temperature was set at 80 °C, with diamond particle size of 45/50, diamond loading of 1 g, magnetic stirring speed of 100 r/min, electroless nickel plating duration of 60 min, and sodium hypophosphite concentration ranging from 12.5 to 20 mL/L. The plating solution parameters with different sodium hypophosphite concentration are presented in Table 7.

#### 5.2.1. The Influence of Sodium Hypophosphite Concentration on the Plating Efficiency

**Figure 7:**
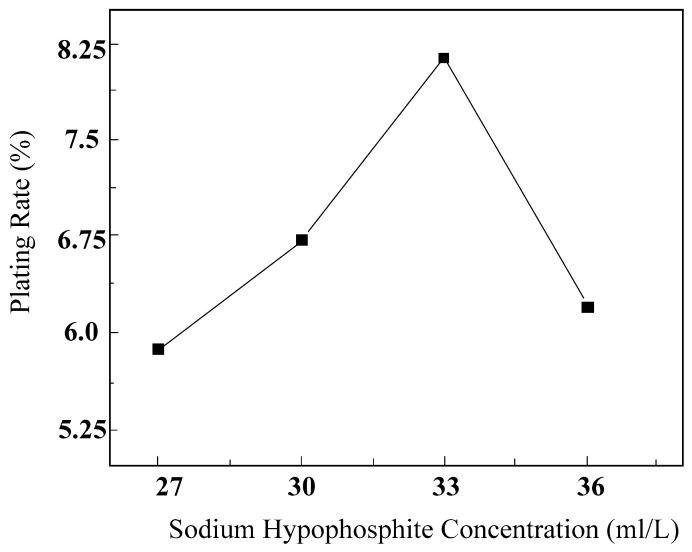
The effect of sodium hypophosphite concentration on plating efficiency.

Figure 7 illustrates the impact of sodium hypophosphite concentration on the surface plating ratio. The graph reveals that when the sodium hypophosphite concentration is too low, the surface plating ratio is comparatively low. This is attributed to the fact that a lower concentration of sodium hypophosphite results in a reduced amount of hydrogen ions (H) in the solution, leading to a decreased number of nickel ions (Ni) being reduced, thereby reducing the surface plating ratio. As the sodium hypophosphite concentration increases, the amount of decomposed hydrogen ions in the solution increases, leading to an increased number of reduced nickel ions, and consequently, the surface plating ratio continuously increases.

When the concentration of sodium hypophosphite in the electroless plating solution is 33 g/L, the surface plating ratio reaches its maximum; however, as the concentration of sodium hypophosphite continues to increase, the surface plating ratio begins to decrease. The primary reason is that when the concentration of sodium hypophosphite is excessively high, it spontaneously decomposes to produce hydrogen gas. The accumulation of hydrogen gas affects the chemical equilibrium of the plating solution, leading to instability and turbidity, which in turn reduces the surface plating ratio.

#### 5.2.2. The Influence of Sodium Hypophosphite Concentration on the Microscopic Morphology of the Coating

**Figure 8:**
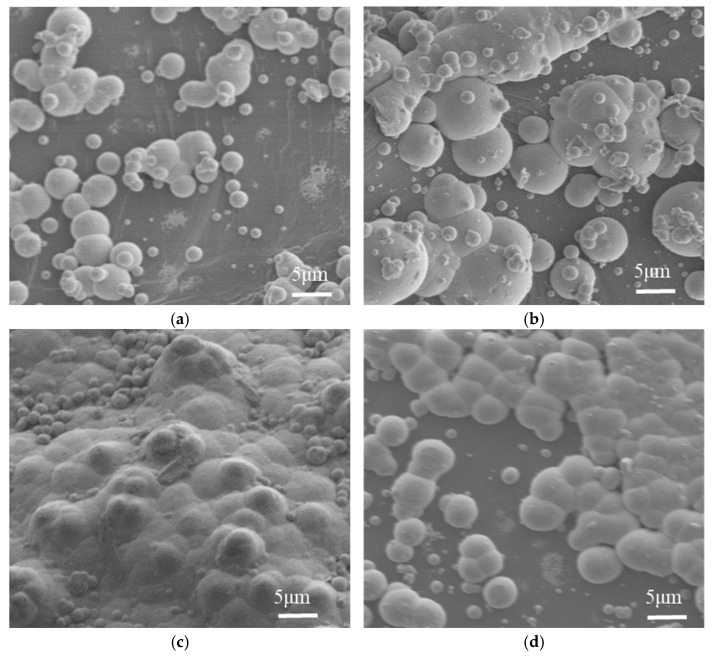
Scanning electron microscope images (×1000) of the microscopic morphology of the coating at different concentrations of sodium hypophosphite. (**a**) Sodium hypophosphite concentration of 27 g/L, (**b**) sodium hypophosphite concentration 30 g/L, (**c**) sodium hypophosphite concentration of 33 g/L, (**d**) sodium hypophosphite concentration of 36 g/L.

Scanning electron microscopy was employed to observe the microtopography of nickel-plated diamond coatings under varying concentrations of sodium hypophosphite, with the results presented in Figure 8. As shown in Figure 8a, when the sodium hypophosphite concentration was 27 g/L, the nickel nodules that formed on the diamond surface were relatively small in size, and some areas remained uncoated. Figure 8b reveals that at a sodium hypophosphite concentration of 30 g/L, the size of the nickel nodules on the coating surface began to increase. Figure 8c demonstrates that when the sodium hypophosphite concentration reached 33 g/L, the diamond surface exhibited the highest number and largest size of nickel nodules, forming a distinct coating. However, as the sodium hypophosphite concentration increased to 36 g/L, as shown in Figure 8d, the diamond surface coating became sparse, and the number of nickel nodules began to decrease.

The primary reason is that during the electroless nickel plating process, nickel nodules tend to form on the diamond surface where catalytic centers are present. In areas without catalytic centers, the low concentration of sodium hypophosphite results in insufficient reduction driving force, preventing the electroless nickel plating reaction from continuing. This leads to significant areas of unplated surface, resulting in uneven plating phenomena. When the concentration of sodium hypophosphite is excessively high, its spontaneous decomposition produces hydrogen gas. The accumulation of hydrogen gas disrupts the chemical equilibrium of the plating solution, causing it to become unstable and turbid. Consequently, the number and size of nickel nodules on the diamond surface decrease, and the plating becomes sparse.

### 5.3. The Influence of Plating Temperature on Electroless Nickel Plating

A single-factor experiment was designed to investigate the influence of plating temperature on the surface plating ratio and the microstructure of electroless nickel-plated diamond. The diamond particle size was selected as 45/50, with a plated diamond loading of 1 g, a magnetic stirring speed of 100 r/min, electroless nickel plating for 60 min, and a plating temperature ranging from 75 to 85 °C, as shown in Table 8.

#### 5.3.1. The Influence of Plating Temperature on the Surface Plating Ratio

**Figure 9:**
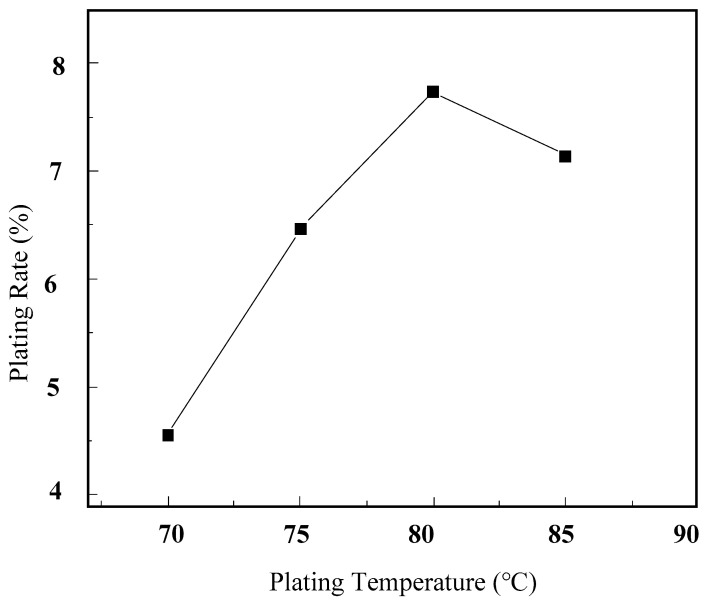
The influence of plating temperature on surface plating ratio.

Figure 9 illustrates the impact of plating temperature on the surface plating ratio. It can be observed from the graph that at a plating temperature of 70 °C, the lower temperature results in reduced ionic activity and diffusion rates, leading to insufficient chemical kinetics. Consequently, there is less hydrogen (H) produced through the decomposition of sodium hypophosphite, which in turn leads to a lower surface plating ratio. Within the temperature range of 70–80 °C, as the plating temperature increases, the surface plating ratio gradually increases.

This is primarily due to the following: as the plating temperature increases, the decomposition of sodium hypophosphite yields more hydrogen, thereby promoting an increase in the surface plating ratio. However, when the temperature continues to rise to 85 °C, the surface plating ratio begins to decrease. This is because as the temperature continues to increase, the chemical kinetic energy increases, and even without the effect of catalytic centers, sodium hypophosphite spontaneously decomposes to produce hydrogen, increasing the reduction rate of Ni^+^. Consequently, the reduced nickel element mainly deposits at the bottom of the beaker rather than on the diamond surface, leading to a decrease in the surface plating ratio.

#### 5.3.2. The Influence of Plating Temperature on the Microstructure of the Coating

**Figure 10:**
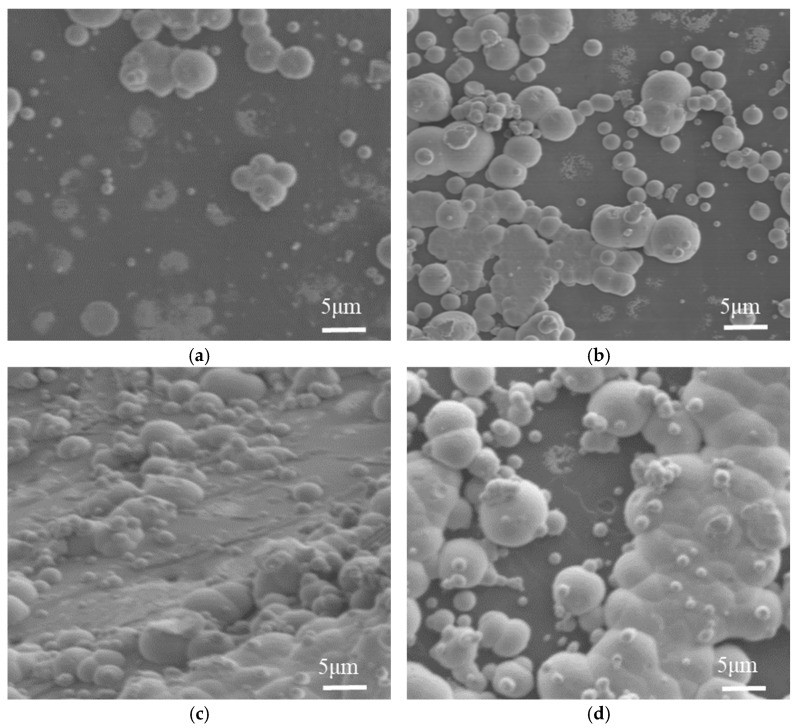
Scanning electron microscope images (×1000) of coating microstructures at different plating temperatures. (**a**) Plating temperature of 70 °C, (**b**) plating temperature of 75 °C, (**c**) plating temperature of 80 °C, (**d**) plating temperature of 85 °C.

The scanning electron microscopy (SEM) was employed to observe the microtopography of the nickel-plated diamond coating surface at different plating temperatures, with the results presented in Figure 10. As observed in Figure 10a, when the plating temperature was at 70 °C, only a small amount of discontinuous nickel nodules was present on the diamond surface. As the plating temperature gradually increased, the number of nickel nodules on the diamond surface gradually increased and their size enlarged. When the plating temperature reached 80 °C, a continuous coating layer was formed on the diamond surface, as observed in Figure 10c. As the plating temperature continued to increase to 85 °C, the coating layer on the diamond surface again exhibited a phenomenon of missed plating, as seen in Figure 10d.

As the plating temperature increases, the chemical kinetics within the plating solution intensify, leading to enhanced ion activity and diffusion rates. Consequently, the decomposition of sodium hypophosphite generates more hydrogen ions, thereby accelerating the growth of nickel nodules and initiating the formation of a coating on the diamond surface. As the plating temperature continues to rise, the chemical kinetic energy increases. Even in the absence of catalytic centers, sodium hypophosphite spontaneously decomposes, releasing hydrogen atoms. This increases the reduction rate of the solution, causing nickel ions to be reduced to elemental nickel and deposited at the bottom of the beaker, ultimately leading to missed plating.

### 5.4. The Influence of Diamond Grain Size on Electroless Nickel Plating

**Figure 11:**
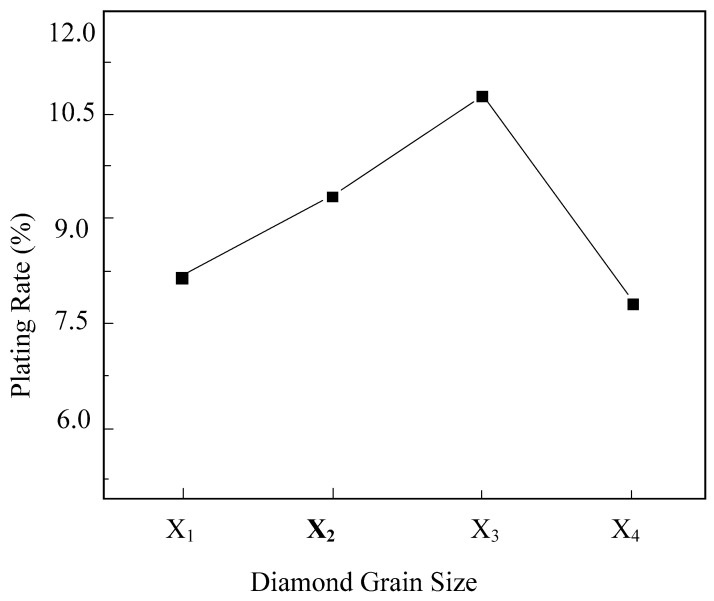
The influence of diamond grain size on the surface plating ratio.

A single-factor experiment was designed to investigate the impact of diamond granularity on the plating efficiency of electroless nickel plating on diamond. The diamond granularities were categorized as X1: 45/50, X2: 70/80, X3: 120/140, and X4: 230/270. Figure 11 illustrates the effect of diamond granularity on the plating efficiency. It is evident from the graph that as the diamond granularity increases, the plating efficiency initially increases and then decreases. At a diamond granularity of 120/140, the plating efficiency reaches 10.75%.

The primary reasons are the increase in diamond grain size and the decrease in grain diameter leading to an increase in the specific surface area of individual diamond particles, thereby enhancing the number of activation centers. During the electroless nickel plating process, the proliferation of these activation centers accelerates the deposition rate of the coating, resulting in an increase in the surface plating ratio. However, as the diamond grain size continues to increase to 230/270, the surface plating ratio begins to decrease. This decline is primarily attributed to the decrease in diamond grain diameter due to the increase in grain size, leading to diamond agglomeration during the electroless nickel plating process. The uneven dispersion of diamonds in the plating solution results in a reduction in the surface plating ratio.

## 6. Conclusions

(1) Silver nitrate was employed as the activator for the pretreatment of diamond surfaces prior to electroless nickel plating. The activation effects of two activation solutions, namely a 2 g/L AgNO_3_ solution and a mixture of a 2 g/L AgNO_3_ solution and 10 mL/L NH_3_·H_2_O, were investigated. The results indicated that the combination of a 2 g/L AgNO_3_ solution and 10 mL/L NH_3_·H_2_O as the activation solution achieved a higher surface plating ratio on electroless nickel-plated diamonds, with a greater number of nickel nodules formed on the plating surface. This demonstrated the feasibility of using silver nitrate as an activator in the pretreatment of diamond surfaces.
(2) An activation solution consisting of AgNO_3_ 2 g/L and NH_3_·H_2_O 10 mL/L was employed for the pretreatment of diamond surfaces, facilitating the investigation of electroless nickel plating on diamonds. The results of orthogonal experiments indicated that the primary and secondary factors affecting the process were in the following order: plating temperature > ammonia concentration > sodium hypophosphite concentration. The findings from single-factor experiments revealed that as the plating temperature, ammonia concentration, and sodium hypophosphite concentration increased, the surface plating ratio of electroless nickel on diamonds, as well as the quantity and size of nickel nodules formed on the diamond surface, exhibited an initial increase followed by a decrease.
(3) Based on the analysis of the experimental results, the optimal process conditions were determined as follows: an ammonia solution concentration of 17.5 mL/L, a hypophosphite concentration of 33 g/L, and a plating temperature of 80 °C. Under these process conditions, the highest deposition rate of electroless nickel on diamonds was achieved, and a continuous and dense coating layer was formed on the diamond surface.

## Figures and Tables

**Table 1:** Chemical Composition of the Activating Solution.

Activation Solution	Chemical Composition
A	AgNO_3_ (2 g/L)
B	AgNO_3_ (2 g/L) + NH_3_·H_2_O (10 mL/L)

**Table 2:** Main components of electroless plating solution.

Components	Medicine	Concentration	Role
Metal salt	Nickel Sulfate	25 g/L	Provision of metal ions
Reducing agent	Sodium Hypophosphite	33 g/L	Reduction in metal ions
	Sodium Citrate	12.5 g/L-	
Complexant	Ammonia solution	17.5 mL/L	Prevent the precipitation of precipitates from the plating solution
	Citric Acid	20 g/L	
Stabilizer	Thiourea	1.4 mg/L	Stable plating solution
Dispersant	Sodium Dodecyl Benzene Sulfonate	1 g/L	Improving the phenomenon of particles

**Table 3:** Orthogonal experiment.

	Factors	A	B	C
		Ammonia Solution Concentration	Sodium Hypophosphite Concentration	Plating Temperature
Levels		(mL/L)	(g/L)	(°C)
1		15	30	75
2		17.5	33	80
3		20	36	85

**Table 4:** Orthogonal experimental schedule and results.

	Factors	Ammonia Solution Concentration	Sodium Hypophosphite Concentration	Plating Temperature	Surface Plating Ratio
Study Number		(mL/L)	(g/L)	(°C)	(%)
1		15	30	75	4.56
2		15	33	80	6.72
3		15	36	85	5.54
4		17.5	30	80	7.74
5		17.5	33	85	7.13
6		17.5	36	75	5.83
7		20	30	85	6.71
8		20	33	75	5.75
9		20	36	80	6.32

**Table 5:** Analysis of the extreme range of surface plating ratios for electroless nickel plating on diamonds.

	Factors	A	B	C
Levels				
R1		5.61	6.33	5.38
R2		6.9	6.53	6.93
R3		6.26	5.89	6.46
range		1.29	0.64	1.55

**Table 6:** Plating solution parameters with different ammonia concentration.

	Factors	Ammonia Solution (mL/L)	Nickel Sulfate (g/L)	Sodium Hypophosphite (g/L)	Citric Acid (g/L)	Sodium Citrate (g/L)	SDBS (g/L)	Thiourea (mg/L)
Number								
1		12.5	25	33	20	12.5	1	1.4
2		15	25	33	20	12.5	1	1.4
3		17.5	25	33	20	12.5	1	1.4
4		20	25	33	20	12.5	1	1.4

**Table 7:** Plating solution parameters with different sodium hypophosphite concentration.

	Factors	Nickel Sulfate (g/L)	Sodium Hypophosphite (g/L)	Citric Acid (g/L)	Sodium Citrate (g/L)	Ammonia Solution (mL/L)	SDBS (g/L)	Thiourea (mg/L)
Number								
1		25	27	20	12.5	17.5	1	1.4
2		25	30	20	12.5	17.5	1	1.4
3		25	33	20	12.5	17.5	1	1.4
4		25	36	20	12.5	17.5	1	1.4

**Table 8:** Plating solution parameters with plating temperature ranging from 75 to 85 °C.

Chemical Composition	Concentration
Nickel Sulfate	25 g/L
Citric Acid	20 g/L
Sodium Citrate	12.5 g/L
Thiourea	1.4 mg/L
SDBS	1 g/L
Ammonia solution	17.5 mL/L
Sodium Hypophosphite	33 g/L

## Data Availability

The original contributions presented in this study are included in the article. Further inquiries can be directed to the corresponding author.

## References

[1] Zeng Z., Yang L., Zeng Q., Lou H., Sheng H., Wen J., Miller D., Meng Y., Yang W., Mao W. (2017). Synthesis of quenchable amorphous diamond. Nat. Commun..

[2] Ding M., Liu Y., Lu X., Tang W. (2019). Effect of Laser Ablation on Microwave Attenuation Properties of Diamond Films. Materials.

[3] Liu D., Zhao J., Lei Y., Wang X., Fu W., Song X., Long W. (2022). Micropatterning of synthetic diamond by metal contact etching with Ti powder. Diam. Relat. Mater..

[4] Kondrin M., Lebed Y., Brazhkin V. (2020). Intrinsic planar defects in diamond and the upper limit on its melting temperature. Diam. Relat. Mater..

[5] Peng J.W., Zhang F.L., Wu Y.X., Zhou Y.M., Tang H.Q. (2023). Wear evolution of metal bond diamond tool in grinding of sapphire. Diam. Relat. Mater..

[6] Zhao X., Li J., Duan L., Tan S., Fang X. (2019). Effect of Fe-based pre-alloyed powder on the microstructure and holding strength of impregnated diamond bit matrix. Int. J. Refract. Met. Hard Mater..

[7] Suzuki T., Konno T. (2014). Improvement in tool life of electroplated diamond tools by Ni-based carbon nanotube composite coatings. Precis. Eng..

[8] Du Q., Wang X., Zhang S., Long W., Zhang L., Jiu Y., Yang C., Zhang Y., Yang J. (2020). Research status on surface metallization of diamond. Mater. Res. Express.

[9] Ahn J., Kim D., Lee J., Chung H., Kim C., Hai H. (2006). Improving the adhesion of electroless-nickel coating layer on diamond powder. Surf. Coat. Technol..

[10] Jia J., Bai S., Xiong D., Xiao J., Yan T. (2020). Enhanced thermal conductivity in diamond/copper composites with tungsten coatings on diamond particles prepared by magnetron sputtering method. Mater. Chem. Phys..

[11] Wei C., Xu X., Wei B., Chen P., Cheng J. (2020). Titanium coating on the surface of diamond particles by a novel rapid low-temperature salt bath plating method. Chem. Phys. Lett..

[12] Das M.K., Liu R., Qin J., Zhang X., Das K., Thueploy A., Limpanart S., Boonyongmaneerat Y., Ma M., Li R. (2017). Effect of electrodeposition conditions on structure and mechanical properties of Ni-W/diamond composite coatings. Surf. Coat. Technol..

[13] Kang Q., He X., Ren S., Zhang L., Wu M., Guo C., Cui W., Qu X. (2013). Preparation of copper–diamond composites with chromium carbide coatings on diamond particles for heat sink applications. Appl. Therm. Eng..

[14] Tian D., Li D., Wang F., Xiao N., Liu R., Gao W., Li N., Li Q., Gao W., Wu G. (2013). A Pd-free activation method for electroless nickel deposition on copper. Surf. Coat. Technol..

[15] Luo L., Lu Z., Huang X., Tan X., Ding X., Cheng J., Zhu L., Wu Y. (2014). Electroless copper plating on PC engineering plastic with a novel palladium free surface activation process. Surf. Coat. Technol..

[16] Shu Z., Wang X. (2012). Environment-friendly Pd free surface activation technics for ABS surface. Appl. Surf. Sci..

[17] Dong Y., He X., Ud-din R., Guo C., Xu L., Huang Y., Qu X. (2011). Fabrication and thermal stability of Ni-P coated diamond powder using electroless plating. Int. J. Min. Met. Mater..

[18] Ahn J., Jin K., Wu J., Hai H., Lee J., Saeng C. (2004). Charateristics of Nickel-Diamond Composite Powders by Eletroless Plating. J. Korean. Powe. Met..

[19] Liu P., Zhu Y. (2015). Interaction Between Fine Diamond Particles in Electroless Nickel Solutions. J. Dispersion Sci. Technol..

[20] Salvaga G., Cavallotti P. (1972). Characteristics of the Reduction of Nickel Alloys with HypopHoslHite. Plating.

[21] Manik B., Tapan K.B., Prasanta S. (2022). Effect of Coating Bath Parameters on Properties of Electroless Nickel-Boron Alloy Coatings. Int. J. Surf. Eng. Interdiscip. Mater. Sci. (IJSEIMS).

